# Influence of Cocoa Flavanols and Procyanidins on Free Radical-induced Human Erythrocyte Hemolysis

**DOI:** 10.1080/17402520512331329514

**Published:** 2005-03

**Authors:** Qin Yan Zhu, Derek D. Schramm, Heidrun B. Gross, Roberta R. Holt, Sun H. Kim, Tomoko Yamaguchi, Catherine L. Kwik-Uribe, Carl L. Keen

**Affiliations:** 1 Department of Nutrition University of California One Shields Avenue Davis CA 95616-8669 USA; 2 Internal Medicine University of California One Shields Avenue Davis CA 95616-8669 USA; 3 Department of Food Service Management and Nutrition Kongju National University Korea; 4 Department of Food Science and Nutrition Nara Women's University Japan; 5 Analytical & Applied Sciences Mars Incorporated Hackettstown NJ USA

## Abstract

Cocoa can be a rich source of antioxidants including the flavan-3-ols, epicatechin and catechin, and their oligomers (procyanidins). While these flavonoids have been reported to reduce the rate of free radical-induced erythrocyte hemolysis in experimental animal models, little is known about their effect on human erythrocyte hemolysis. The major objective of this work was to study the effect of a flavonoid-rich cocoa beverage on the resistance of human erythrocytes to oxidative stress. A second objective was to assess the effects of select purified cocoa flavonoids, epicatechin, catechin, the procyanidin Dimer B2 and one of its major metabolites, 3ʹ-*O*-methyl epicatechin, on free radical-induced erythrocyte hemolysis *in vitro*. Peripheral blood was obtained from 8 healthy subjects before and 1, 2, 4 and 8 h after consuming a flavonoid-rich cocoa beverage that provided 0.25 g/kg body weight (BW), 0.375 or 0.50 g/kg BW of cocoa. Plasma flavanol and dimer concentrations were determined for each subject. Erythrocyte hemolysis was evaluated using a controlled peroxidation reaction. Epicatechin, catechin, 3ʹ-*O*-methyl epicatechin and (-)-epicatechin-(4β > 8)epicatechin (Dimer B2) were detected in the plasma within 1 h after the consumption of the beverage. The susceptibility of erythrocytes to hemolysis was reduced significantly following the consumption of the beverages. The duration of the lag time, which reflects the capacity of cells to buffer free radicals, was increased. Consistent with the above, the purified flavonoids, epicatechin, catechin, Dimer B2 and the metabolite 3ʹ-*O*-methyl epicatechin, exhibited dose-dependent protection against AAPH-induced erythrocyte hemolysis at concentrations ranging from 2.5 to 20 μM. Erythrocytes from subjects consuming flavonoid-rich cocoa show reduced susceptibility to free radical-induced hemolysis (*p* < 0.05).


*Clinical & Developmental Immunology*, March 2005; 12(1): 27–34

### Influence of cocoa flavanols and procyanidins on free radical-induced human erythrocyte hemolysis

QIN YAN ZHU[1], DEREK D. SCHRAMM[1], HEIDRUN B. GROSS[1], ROBERTA R. HOLT[1], SUN H. KIM[3], TOMOKO YAMAGUCHI[4], CATHERINE L. KWIK-URIBE[5], & CARL L. KEEN[1,2]

[1]*Department of Nutrition, University of California, One Shields Avenue, Davis, CA 95616-8669,* [2]*Internal Medicine, University of California, One Shields Avenue, Davis, CA 95616-8669,* [3]*Department of Food Service Management and Nutrition, Kongju National University, Republic of Korea,* [4]*Department of Food Science and Nutrition, Nara Women's University, Japan, and* [5]*Analytical & Applied Sciences, Mars Incorporated, Hackettstown, NJ, USA*

**Abstract**

Cocoa can be a rich source of antioxidants including the flavan-3-ols, epicatechin and catechin, and their oligomers (procyanidins). While these flavonoids have been reported to reduce the rate of free radical-induced erythrocyte hemolysis in experimental animal models, little is known about their effect on human erythrocyte hemolysis. The major objective of this work was to study the effect of a flavonoid-rich cocoa beverage on the resistance of human erythrocytes to oxidative stress. A second objective was to assess the effects of select purified cocoa flavonoids, epicatechin, catechin, the procyanidin Dimer B2 and one of its major metabolites, 3′-*O*-methyl epicatechin, on free radical-induced erythrocyte hemolysis *in vitro*. Peripheral blood was obtained from 8 healthy subjects before and 1, 2, 4 and 8 h after consuming a flavonoid-rich cocoa beverage that provided 0.25 g/kg body weight (BW), 0.375 or 0.50 g/kg BW of cocoa. Plasma flavanol and dimer concentrations were determined for each subject. Erythrocyte hemolysis was evaluated using a controlled peroxidation reaction. Epicatechin, catechin, 3′-*O*-methyl epicatechin and (-)-epicatechin-(4β > 8)-epicatechin (Dimer B2) were detected in the plasma within 1 h after the consumption of the beverage. The susceptibility of erythrocytes to hemolysis was reduced significantly following the consumption of the beverages. The duration of the lag time, which reflects the capacity of cells to buffer free radicals, was increased. Consistent with the above, the purified flavonoids, epicatechin, catechin, Dimer B2 and the metabolite 3′-*O*-methyl epicatechin, exhibited dose-dependent protection against AAPH-induced erythrocyte hemolysis at concentrations ranging from 2.5 to 20 μM. Erythrocytes from subjects consuming flavonoid-rich cocoa show reduced susceptibility to free radical-induced hemolysis ($p < 0.05$).

**Keywords:** *Cocoa, flavanol, membrane oxidation, erythrocyte, (+)-catechin, 3′-O-methyl epicatechin*

### Introduction

Flavonoids are a diverse group of polyphenolic compounds occurring in fruit, vegetables, tea and red wine. Diets rich in these compounds have been associated with a reduced risk for cardiovascular disease (Knekt et al. 2002, Kris-Etherton et al. 2004, Mennen et al. 2004, Morton et al. 2000, Sagara et al. 2004, Sesso et al. 2003). Depending on their processing, cocoa and chocolate can be rich sources of flavonoids, particularly the subclass of oligomeric flavonoids known as procyanidins (Adamson et al. 1999, Sanchez-Rabaneda et al. 2003, Steinberg et al. 2003) (Figure 1). Flavanols and procyanidins isolated from cocoa and chocolate have received considerable attention over the last several years as potential cardiovascular protective agents with putative positive effects on platelet function, blood pressure regulation, nitric oxide production, oxidative defense and the immune system (Heiss et al. 2003, Maron, 2004; Fisher et al. 2003; Pearson et al. 2002, Sadik et al. 2003, Taubert et al. 2003, Wan et al. 2001).

Correspondence: Carl L. Keen, Department of Nutrition, University of California, One Shields Avenue, Davis, CA 95616-8669. Tel: 1 530 752 6331. Fax: 1 530 752 8966. E-mail: clkeen@ucdavis.edu

ISSN 1740-2522 print/ISSN 1740-2530 online © 2005 Taylor & Francis Group Ltd.
DOI: 10.1080/17402520512331329514

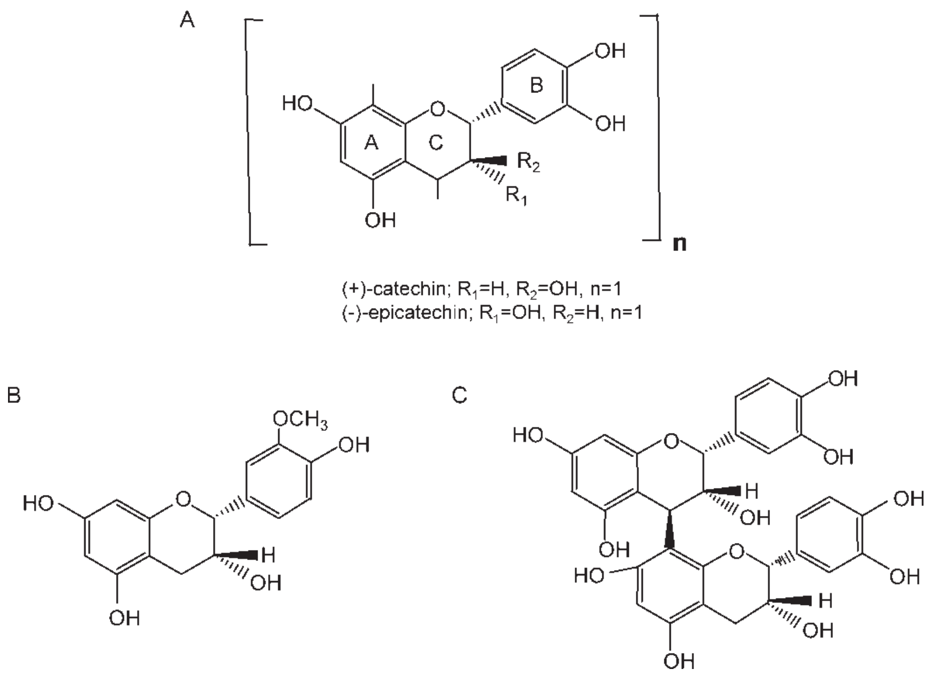

Figure 1. Chemical structures of (A) monomer and procyanidin oligomers, $n = 1$ (monomer), 2 (dimer) to 10 (decamer); (B) 3′-*O*-methyl epicatechin; (C) Dimer B2 (epicatechin-(4β > 8)-epicatechin.

These compounds can modulate the actions of oxidant-responsive transcription factor NF-κB (Mackenzie et al. 2004), and diets rich in cocoa flavonoids may be associated with reductions in DNA oxidative damage (Orozco et al. 2003).

Excessive oxidative damage to cellular membranes may contribute to the development of many degenerative diseases, including certain cancers and cardiovascular diseases (Cooke et al. 2003, Hensley et al. 2000, Kris-Etherton et al. 2004, Morena et al. 2000, Morton et al. 2000). Erythrocytes are especially susceptible to oxidation due to their high content of polyunsaturated lipids, their rich oxygen supply and the presence of transition metals such as iron and copper (Delmas-Beauvieux et al. 1995, Mennen et al. 2004). Reactive oxygen species (ROS) generated in the plasma, cytosol, or cell membrane can attack erythrocyte membranes, compromise their integrity, and induce the oxidation of lipids and protein resulting in hemolysis (Bracci et al. 2002, Chan et al. 1999, Delmas-Beauvieux et al. 1995, Niki et al. 1988). Given their function, and their susceptibility to oxidative damage, investigators have used erythrocytes, and free radical initiators such as 2,2′-azo-bis (2-amidinopropane) dihydrochloride (AAPH) and UVB as models for studying biomembrane oxidative damage (Carini et al. 2000, Chan et al. 1999, Liu et al. 2002, Mabile et al. 2001, Niki et al. 1988, Zhu et al. 2002). We recently studied the inhibitory effects of flavanoid-rich cocoa extract on rat erythrocyte hemolysis, and found that hemolysis resistance was enhanced following the feeding of this extract ($p < 0.05$) (Zhu et al. 2002). While these results are provocative, the differences in flavonoid metabolism between rats and humans (Natsume et al. 2003), and the fact that an extract rather than a food per se was used in the above study preclude a direct extrapolation of these results to humans.

In the present work, we evaluated the effects of a flavanol-rich cocoa on the susceptibility of human erythrocytes to hemolysis. We also assessed the ability of select purified cocoa flavonoids (epicatechin and catechin, Dimer B2), and one of the major metabolites of epicatechin, 3′-*O*-methyl epicatechin, to protect against human erythrocyte hemolysis, *in vitro*.

### Subjects and methods

### *Subjects and clinical study design*

Eight healthy adult male subjects were recruited from the general population at the University of California at Davis. All subjects signed informed consent forms prior to the study in accordance with the guidelines of the Human Subjects Review Committee of the University of California, Davis.

In a cross-over study design, the subjects consumed a low polyphenolic control meal of bread and water (1.5 g/kg and 10 ml/kg subject body weight, respectively), followed by a low [0.25 g/kg body weight (BW)], medium (0.375 g/kg BW), or high flavonoid-rich cocoa beverage (0.50 g/kg BW), with a one week washout period between doses. One gram of the cocoa provided 12.2 mg monomers (epicatechin and catechin), 9.7 mg dimers (consisting of two monomer units) and 28.2 mg of the longer procyanidins (trimers through decamers built upon monomer units).

The mean age of the subjects was 26 ± 2 y (range: 19–31), the mean body weight was 79.1 ± 5.7 kg, the average height was 1.80 ± 0.05 m and the mean body mass index (in kg/m$^2$) was 24 ± 1. All subjects were apparently healthy.

On the day before the experiment, subjects were asked to refrain from consuming phenolic-rich foods (e.g. tea, wine, fruits and vegetables). They were asked to fast (no food or drink, except water) for approximately 10 h prior to the experiment. On the day of the study, blood was drawn by venipuncture to establish baseline values. Within 15 min after the baseline blood draw, subjects were given bread [1.5 g/kg BW] and water (10 ml/kg BW) in the first visit (week 1). Venous blood samples were then obtained at 1, 2, 4 and 8 h after the initial baseline draw. Blood was drawn into evacuated tubes containing sodium heparin as an anticoagulant, and the plasma was then separated by low-speed centrifugation (3,000 × *g*) at 4°C for 10 min, and stored at −80°C until further analysis. Weeks 2, 3 and 4 were the same except for doses of cocoa: subjects received 0.250 g/kg BW cocoa at week 2, 0.375 g/kg BW at week 3 and 0.50 g/kg BW at week 4 (Cocoapro; Mars Inc, Hackettstown, NJ).

### *Erythrocytes hemolysis*

Immediately after the blood draw, erythrocytes were separated from the plasma and buffy coat by centrifugation at 3,000 × *g* for 10 min. The crude erythrocytes were washed three times with 5 vol of phosphate buffered saline, pH = 7.4 (PBS, GIBCO BRL, Invitrogen Life Technologies, Carlsbad, CA). During the last wash, the erythrocytes were centrifuged at 3,000 × *g* for 20 min to obtain a packed cell preparation. The packed erythrocytes were then suspended in 4 vol of PBS solution.

The method of Miki et al. (1987) was used to determine radical mediated hemolysis, with minor modifications (Zhu et al. 2002). Briefly, washed erythrocytes were resuspended in PBS at 10% hematocrit, and after the addition of 200 mM AAPH (1:1), the reaction mixture was incubated at 37°C. Samples of the reaction mixture were obtained and centrifuged every 20 min. The supernatant fraction was then discarded, and the pellet lysed with 3 ml of distilled water. Hemoglobin concentration in the supernatant fraction after centrifugation was determined by measuring the absorbance at 540 nm. Erythrocytes incubated without AAPH and treated similarly were used to measure the total hemoglobin content of the cells. The percentage of hemolysis induced by AAPH was calculated by the equation:

$$\%\text{Hemolysis} = [1 - A_{\text{sample}}/A_{\text{total}}] \times 100 \qquad (1)$$

Where: $A_{\text{sample}}$ is the absorbance of the test sample collected every 20 min from the reaction mixture, and $A_{\text{total}}$ is the total hemoglobin content of the cells.

### *In vitro study of epicatechin, catechin, Dimer B2 and 3′-O-methyl epicatechin*

Epicatechin, catechin and Dimer B2 purified from cocoa were provided by Mars Incorporated (Cocoapro$^{\text{TM}}$, Hackettstown, NJ) (Adamson et al. 1999). The 3′-*O*-methylated conjugate of epicatechin (Figure 1B) was synthesized using a mixture of 250 mg (-)-epicatechin, 500 mg $K_2CO_3$ and 1 ml methyl iodide in 20 ml acetone, which was placed in an ultrasonic bath for 2.5 h. The 3′-*O*-methylated conjugates were purified by semi-preparative HPLC and the positions of the methyl groups were confirmed as described by Donovan et al. (1999). The erythrocyte hemolysis was conducted according to methods previously described (Zhu et al. 2002). Briefly, 1 ml of the erythrocyte suspension was mixed with 1 ml of PBS solution containing varying amounts of cocoa flavonoids and 3′-*O*-methyl epicatechin (7.5–60 μM). About 1 ml of 200 mM AAPH in PBS was then added to the mixture. The reaction mixture was shaken gently while being incubated at 37°C for 3 h. After incubation, the reaction mixture was diluted with 8 vol of PBS and centrifuged at 3,000 × *g* for 5 min. The absorbance (A) of the supernatant fraction at 540 nm was recorded in a Beckman DU 640 spectrophotometer. Percent inhibition was calculated as described before (Zhu et al. 2002). L-Ascorbic acid was used as a positive control (2.5–20 μM).

### *HPLC analysis of epicatechin and catechin*

Plasma samples were extracted as previously described by Holt et al. (2002). After first treating the plasma with a mixed solution of β-glucuronidase and sulfatase (Type HP-2, from *Helix pomatia*; Sigma Chemical, St. Louis, MO) to hydrolyze glucuronide, sulfate or sulfoglucuronide conjugates to the nonconjugated free form of epicatechin and catechin. The resulting solution was filtered with a 0.22 μm Millipore Ultrafree-MC low binding Durapore centrifugal filter (Millipore, Bedford, MA). 50 μl of the filtered solution was analyzed for catechin, epicatechin and procyanidins by reversed-phase HPLC with coulometric multiple-array detection.

Chromatography was carried out as described by Holt et al. (2002) using a HP 1100 HPLC system equipped with Chemstation software and a quaternary pump (Hewlett Packard, Wilmington, DE), and an ESA CoulArray 5600 coulometric electrochemical array detector (ESA, Chelmsford, MA). Epicatechin and catechin peak identification at +150 mV was based on co-elution with authentic standards and quantified using external standards.

### *Statistics*

Data were analyzed using analysis of variance (ANOVA). Between treatment means were compared by independent *t*-test. The significance level was set at 0.05, and analysis was performed using routines available in Statview for Windows (v 5.0.1. SAS, Cary, NC). Results are expressed as means ± SEM.

### Results and discussion

Erythrocytes contain high concentrations of polyunsaturated fatty acids, molecular oxygen and ferrous ions in the ligand state. Hence, it might be expected that erythrocytes would be highly vulnerable to oxidative stress. However, these cells possess an efficient antioxidant system that renders them exceptionally resistant to peroxidation when radicals are produced within the cell (Niki et al. 1988, Miki et al. 1987). The present work investigated the peroxidability of the erythrocyte membrane, in which free radicals were generated by an exogenous source. The peroxidation of erythrocyte membranes can be studied with a variety of agents, including hydrogen peroxide, dialuric acid, xanthine oxidase and organic hydroperoxides (Zhu et al. 2002, Miki et al. 1987), but AAPH offers some specific advantages. Azo compounds, such as AAPH, generate free radicals by their unimolecular thermal decomposition in the aqueous region, and these radicals quickly react with oxygen, producing peroxyl radicals that are able to attack the erythrocyte membrane. The rate of generation of free radicals can be controlled and measured by adjusting the concentration of the initiator. Further, the hemolysis induced by AAPH provides a model for studying oxidative membrane damage, when generation of free oxygen radicals is initiated extracellularly (Zhu et al. 2002, Miki et al. 1987).

An example of the time-dependent rate of AAPH induced erythrocyte hemolysis is given in Figure 2. Sigmoid curves were obtained allowing for the calculation of several quantitative parameters. A simple index for comparing the relative response to peroxidative stress is the time required to achieve 50% hemolysis (A). Another index is lag time (B), defined as the interval (min) between the intercept of the linear least-square slope of the curve with the initial absorbance. This reflects the capacity of the cell to buffer peroxyl radicals. The maximum amount of hemolysis (C) and the time necessary to reach this (D) were also obtained (Sugiyama et al. 1993).

The present study investigated the susceptibility of human erythrocytes to peroxidation after the consumption of a flavonoid-rich cocoa beverage. A typical example of the time-dependent erythrocyte hemolysis curves from one subject who consumed 0.25 g/kg BW of the cocoa beverage is depicted in Figure 2 (Table I gives the means for all the subjects). All three doses of the beverages were effective in protecting against hemolysis. We observed that within 1 h after the subjects consumed the medium dose of cocoa beverage (0.375 g/kg BW), the time necessary for

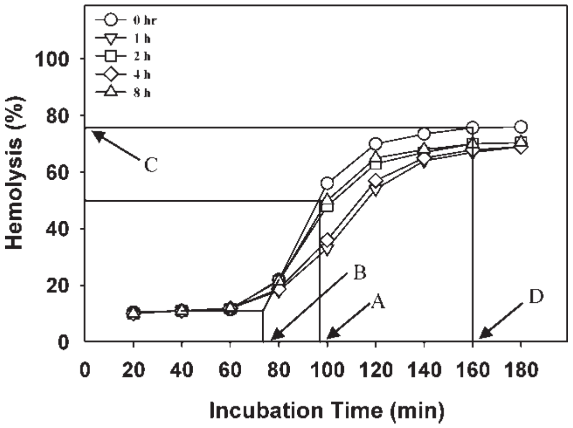

Figure 2. Time-dependent hemolysis of erythrocytes from one subject at baseline, and 1, 2, 4 and 8 h after receiving 0.25 g/kg BW of a cocoa beverage. The reaction was induced by 200 mM AAPH. Arrows indicate the parameters that were calculated: (A) 50% hemolysis; (B) lag time; (C) maximal hemolysis; (D) time at which maximum hemolysis is reached.

peroxyl radicals to hemolyze half of the erythrocytes (Hem 50) increased from 100 ± 4.6 to 120.7 ± 4.3 min. When subjects were given the lower (0.25 g/kg BW) and higher (0.50 g/kg BW) cocoa doses, Hem 50 was significantly increased to 106.5 ± 3.2 and 108.8 ± 3.1 min, respectively, 2 h after cocoa consumption. A similar pattern was found for changes in the lag time and maximum hemolysis (see Table I). Compared to the low (0.25 g/kg) and high (0.5 g/kg BW), the medium dose (0.375 g/kg BW) exhibited faster and stronger inhibition in almost all parameters. The reasons for this inconsistency are obscure, but may result from individual differences; i.e. the fact that sigmoid hemolysis curves vary across subjects. Unpublished data in our laboratory shows that the medium dose has a stronger inhibitory effect upon epinephrine-stimulated platelet activation than either low or high doses. The failure of the *ex vivo* hemolysis to exhibit dose-dependent effects *in vitro* is not known. It may be that the *ex vivo* hemolysis inhibition gradually saturates. Another equally plausible supposition is that the consumption of 3 doses of cocoa powder increases caffeine consumption in a dose-dependent manner. It has been reported (Lecomte and Boivin 1981) that caffeine and other methylxanthines inhibit human erythrocyte phosphatidyl inositol kinase, thymidine kinase and cAMP-independent protein kinase, thus inhibiting protein phosphorylation. As spectrin phosphorylation is necessary for the equilibrium of the erythrocyte cytoskeleton, the caffeine content in the high dose of cocoa could have led to the instability of the erythrocyte membrane, resulting in hemolysis.

It has been well documented that flavonoids from different sources including grape seed extract (Carini et al. 2000, Zou et al. 2001), cocoa

Table I. Parameters measured from the hemolysis curves of erythrocytes.

| | 0 h | 1 h | 2 h | 4 h | 8 h |
|---|---|---|---|---|---|
| Hem 50 (min), control | 100.0 ± 6.5$^{a,1}$ | 97.2 ± 4.2$^{a,1}$ | 95.1 ± 5.6$^{b,1}$ | 102.8 ± 3.2$^{a,1}$ | 101.1 ± 4.2$^{a,1}$ |
| Low dose | 100.0 ± 4.1$^{a,1}$ | 100.0 ± 2.7$^{a,1}$ | 106.5 ± 3.2$^{b,2}$ | 114.0 ± 3.7$^{c,2}$ | 120.3 ± 4.2$^{d,2}$ |
| Medium dose | 100.0 ± 4.6$^{a,1}$ | 120.7 ± 4.3$^{b,2}$ | 106.7 ± 4.2$^{c,2}$ | 116.5 ± 3.2$^{b,2}$ | 104.1 ± 3.1$^{c,1}$ |
| High dose | 100.0 ± 3.4$^{a,1}$ | 106.7 ± 3.7$^{b,1}$ | 108.8 ± 3.1$^{b,2}$ | 109.8 ± 2.9$^{b,2}$ | 105.3 ± 2.1$^{b,1}$ |
| Lag Time (min), control | 100.0 ± 7.9$^{a,1}$ | 97.0 ± 7.8$^{a,1}$ | 94.3 ± 5.7$^{b,1}$ | 95.7 ± 5.7$^{b,1}$ | 98.1 ± 5.6$^{a,1}$ |
| Low dose | 100.0 ± 6.1$^{a,1}$ | 99.3 ± 4.9$^{a,1}$ | 104.2 ± 3.5$^{b,2}$ | 103.6 ± 3.1$^{a,2}$ | 107.8 ± 3.1$^{b,2}$ |
| Medium dose | 100.0 ± 4.1$^{a,1}$ | 104.9 ± 5.5$^{b,2}$ | 102.3 ± 5.6$^{a,2}$ | 104.2 ± 3.4$^{b,2}$ | 101.5 ± 4.1$^{a,1}$ |
| High dose | 100.0 ± 4.3$^{a,1}$ | 101.5 ± 3.3$^{a,2}$ | 102.5 ± 3.5$^{a,2}$ | 103.2 ± 3.0$^{a,2}$ | 101.1 ± 3.9$^{a,1}$ |
| Hem. max (%), control | 100.0 ± 4.7$^{a,1}$ | 100.9 ± 4.8$^{a,1}$ | 102.1 ± 2.0$^{a,1}$ | 103.4 ± 7.5$^{a,1}$ | 103.6 ± 4.4$^{a,1}$ |
| Low dose | 100.0 ± 3.1$^{a,1}$ | 99.6 ± 3.5$^{a,1}$ | 96.9 ± 2.0$^{a,1}$ | 92.7 ± 4.5$^{b,1}$ | 89.2 ± 2.1$^{b,2}$ |
| Medium dose | 100.0 ± 6.8$^{a,1}$ | 88.2 ± 2.5$^{b,2}$ | 92.1 ± 2.5$^{b,2}$ | 87.9 ± 2.8$^{b,2}$ | 92.4 ± 1.7$^{b,2}$ |
| High dose | 100.0 ± 3.6$^{a,1}$ | 98.5 ± 2.3$^{a,1}$ | 89.1 ± 4.2$^{b,2}$ | 89.9 ± 3.9$^{b,2}$ | 99.1 ± 2.3$^{a,1}$ |
| Time of max hemolysis (min), control | 100.0 ± 3.9$^{a,1}$ | 91.2 ± 3.8$^{b,1}$ | 95.5 ± 2.8$^{b,1}$ | 102.5 ± 4.3$^{a,1}$ | 100.6 ± 3.0$^{a,1}$ |
| Low dose | 100.0 ± 3.9$^{a,1}$ | 101.2 ± 4.1$^{a,2}$ | 109.6 ± 4.5$^{b,2}$ | 114.3 ± 3.0$^{c,2}$ | 116.5 ± 5.1$^{c,2}$ |
| Medium dose | 100.0 ± 2.7$^{a,1}$ | 111.0 ± 4.9$^{b,2}$ | 101.9 ± 4.1$^{a,1}$ | 106.9 ± 1.8$^{c,1}$ | 105.3 ± 4.3$^{c,1}$ |
| High dose | 100.0 ± 0.9$^{a,1}$ | 110.4 ± 3.1$^{b,2}$ | 111.7 ± 3.8$^{b,1}$ | 108.1 ± 4.0$^{b,1}$ | 105.7 ± 3.7$^{b,1}$ |

$^{a,b,c,d}$ means in the same row with different superscripts differ significantly at $p < 0.05$. $^{1,2}$ means for the different doses at each time point with different superscripts differ significantly from their respective controls at $p < 0.05$. Data are expressed as mean ± SEM of $n = 8$. *Hem 50: the time required to achieve 50% hemolysis; Lag time: the interval between the intercept of the linear least-square slope of the curve with the initial absorbance; Hem. max (%): the maximum amount of hemolysis; Time of max hemolysis: the time necessary to reach the maximum hemolysis; Low dose: 0.25 g/kg BW of a cocoa beverage; Medium dose: 0.375 g/kg BW of a cocoa beverage; High dose: 0.50 g/kg BW of a cocoa beverage.

(Zhu et al. 2002), green tea (Zhang et al. 1997) and pine bark extract (Sharma et al. 2003) can offer protection against AAPH-induced and UVB-induced erythrocyte damage *in vitro*. The cocoa flavanols (epicatechin, catechin), Dimer B2 and 3′-*O*-methyl epicatechin all provided protective effects ($p < 0.001$) against free radical-induced hemolysis in a dose-dependent manner (2.5 μM–20 μM). The level of protection exceeded that of ascorbic acid (Figure 3). It is interesting to note that both epicatechin and catechin can significantly protect human erythrocytes from free radical-induced hemolysis, even at a concentration as low as 2.5 μM. While the

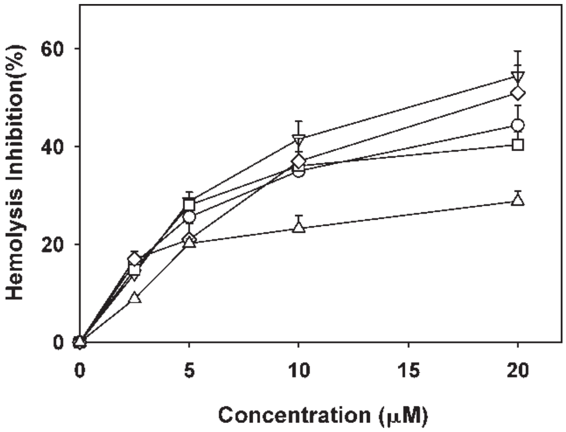

Figure 3. The protective effects of epicatechin (∇-∇), catechin (□-□), Dimer B2 (◇-◇), and 3′-*O*-methyl epicatechin (□-□), expressed as percent inhibition of erythrocyte hemolysis. Ascorbic acid (Δ-Δ) was used as a reference compound. Data are expressed as means ± SEM of $n = 5$–6.

concentrations of epicatechin and catechin in human plasma reached 2.86 μM and 210 nm, respectively (Figure 4), after the consumption of a high dose of cocoa (0.50 g/kg BW), these were sufficient to protect human blood against *in vivo* oxidative damage. Also, the other bioavailable forms of flavonoids (such as Dimer B2) (Baba et al. 2002, Holt et al. 2002, Zhu et al. 2002) and metabolites (such as 3′-*O*-methyl epicatechin) (Day et al. 1998, Kuhnle et al. 2000) known to be present after cocoa consumption would also contribute to this protective activity of cocoa.

Previous studies reported that procyanidins have a stronger protective effect than their monomeric units *in vitro* (Lotito et al. 2000, Arteel et al. 1999, Takahashi et al. 1999, Zhu et al. 2002). This is different from the present result in which epicatechin demonstrated stronger protection than Dimer B2. The reason for this inconsistency is unknown. One of the possible reasons is that in the present *in vitro* study, individual compounds were used, while in the previous studies, a mixture of monomers (epicatechin and catechin) and a mixture of dimers [(Dimer B2 and Dimer B5 (epicatechin-(4β > 6)-epicatechin)] were compared (Zhu et al. 2002). Preliminary work in our lab shows that Dimer B5 has more antioxidant activity than Dimer B2 (unpublished data).

The protective effect of flavonoids toward the erythrocyte membrane has been attributed to their antioxidant capacities. But the results from *in vitro* studies cannot be directly extrapolated to the human situation because the flavonoids applied to those systems are not, in general, the forms circulating *in vivo* after absorption. Flavonoids are generally modified on absorption *in vivo*, and recent studies

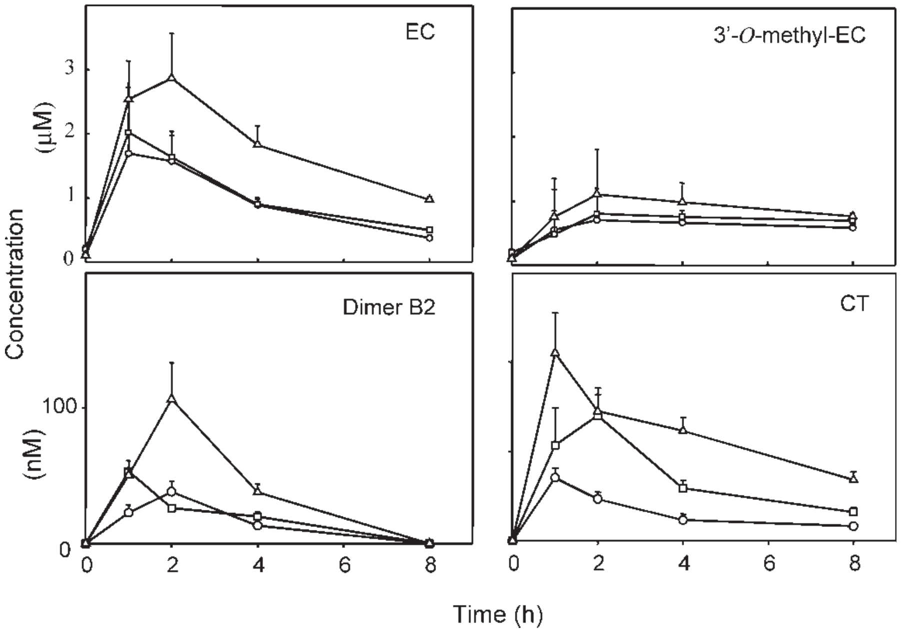

Figure 4. Changes in plasma concentrations of (-)-epicatechin (EC), (+)-catechin (CT), 3′-*O*-methyl epicatechin and (-)-epicatechin-(4β > 8)-epicatechin (Dimer B2) in human subjects after they consumed low-dose (0.25 g/kg BW, O-O), medium dose (0.375 g/kg BW, □-□), and high dose (0.50 g/kg BW, Δ-Δ) flavonoid-rich cocoa beverage. Data are expressed as means ± SEM of $n = 8$.

have shown that they are extensively metabolized, initially during transfer across the small intestine and secondly by the liver, to a variety of conjugates and metabolites. For example, absorption through the small intestine promotes deglycosylation and glucuronidation, as well as *O*-methylation of catechol-containing phenolics (Day et al. 1998, Kuhnle et al. 2000). Because the catechol-containing flavonoid structures are known to be powerful H-donating antioxidants, we also studied the influence of catechol methylation on the antioxidant activity of epicatechin, in the form of 3′-*O*-methyl epicatechin. We observed that the individual flavonoids and 3′-*O*-methyl epicatechin provided similar protection against erythrocyte hemolysis *in vitro* at concentrations ranging from 2.5 to 20 μM (Figure 3). It is interesting to note that both epicatechin, and its metabolite, 3′-*O*-methyl epicatechin, can significantly protect human erythrocytes from free radical-induced hemolysis, even at a concentration as low as 2.5 μM.

The mechanism(s) by which cocoa flavanols and procyanidins protect erythrocytes from hemolysis have not been characterized. It is possible that the consumption of cocoa spares the use of other antioxidants (e.g. ascorbic acid, α-tocopherol), and these in turn afford protection to the cell membrane. Flavonoids have been shown to spare, maintain, or regenerate α-tocopherol and other antioxidants by donating hydrogen to α-tocopherol radicals (Zhu et al. 1999). Another possibility is that flavonoids stabilize the erythrocyte membrane by suppressing the loss of glycolytic intermediates, such as intracellular ATP (adenosine triphosphate) and 2,3-DPG (2,3-diphosphoglycerate) (Moriguchi et al. 2001). Bombardelli et al. (1989) has reported specific localization of procyanidins within the phospholipid bilayer. This suggests the possibility of a preferential binding with membrane phospholipids, very likely via a complexation mechanism, via electrostatic interactions between the nucleophilic phenol groups of oligomeric catechins and the cationic polar heads of phospholipids. This interpretation is supported by NMR studies with procyanidins and other polyphenols and soybean phosphatidylcholine (Bombardelli et al. 1989). Recently, Verstraeten et al. (2003) reported that flavanols and procyanidins interact with membrane phospholipids through hydrogen bonding to the polar head groups of phospholipids. As a consequence, these compounds can accumulate at the membrane surface, both outside and inside the cell. Through this kind of interaction, flavanols and procyanidins might act to maintain membrane integrity by reducing the access of deleterious molecules to the hydrophobic region of the bilayer, including those that can affect membrane rheology as well as those that induce oxidative damage to the membrane components.

In summary, the consumption of a flavonoid-rich cocoa can be associated with a reduction in the rate of erythrocyte hemolysis in human adults. In erythrocytes obtained from healthy adult subjects, lag time and maximal hemolysis were increased for at least 2 h following consumption of the beverage. *In vitro* tests indicate that cocoa flavanols and metabolites possess strong antioxidative activity, resulting in a significant decrease in erythrocyte hemolysis. Additional research is needed to characterize the membrane binding and membrane action of catechin, epicatechin and the procyanidins.

### Acknowledgements

We would like to thank J. L. Ensunsa, T. Y. Momma, M. B. Karim and H. R. Schrader for their technical support. This work was supported in part by the National Institutes of Health (DK-35747), and by a grant from Mars Incorporated.

### References

Adamson GE, Lazarus SA, Mitchell AE, et al. 1999. HPLC method for the quantification of procyanidins in cocoa and chocolate samples and correlation to total antioxidant capacity. J Agric Food Chem 47:4184–4188.

Arteel GE, Sies H. 1999. Protection against peroxynitrite by cocoa polyphenol oligomers. FEBS Lett 462:167–170.

Baba S, Osakabe N, Natsume M, Terao J. 2002. Absorption and urinary excretion of procyanidin B2 [epicatechin-(4beta-8)-epicatechin] in rats. Free Radic Biol Med 33:142–148.

Bombardelli E, Curri SB, Della LR, Del NP, Tubaro A, Gariboldi P. 1989. Complex between phospholipids and vegetable derivatives of biological interest. Fitoterapia LX (suppl. 1):1–9.

Bracci R, Perrone S, Buonocore G. 2002. Oxidant injury in neonatal erythrocytes during the perinatal period. Acta Paediatr Suppl 91:130–134.

Carini M, Aldini G, Bombardelli E, Morazzoni P, Facino RM. 2000. UVB-induced hemolysis of rat erythrocytes: protective effect of procyanidins from grape seeds. Life Sci 67:1799–1814.

Chan AC, Chow CK, Chiu D. 1999. Interaction of antioxidants and their implication in genetic anemia. Proc Soc Exp Biol Med 222:274–282.

Cooke MS, Evans MD, Dizdaroglu M, Lunec J. 2003. Oxidative DNA damage: mechanisms, mutation, and disease. FASEB J 17:1195–1214.

Day AJ, DuPont MS, Ridley S, et al. 1998. Deglycosylation of flavonoid and isoflavonoid glycosides by human small intestine and liver beta-glucosidase activity. FEBS Lett 436:71–75.

Delmas-Beauvieux MC, Peuchan E, Dumon MF. 1995. Relationship between red blood cell antioxidant enzymatic system status and lipoperoxidation during the acute phase of malaria. Clin Biochem 28:163–169.

Donovan JL, Luthria DL, Stremple P, Waterhouse AL. 1999. Analysis of (+)-catechin, (-)-epicatechin and their 3′- and 4′-*O*-methylated analogs. A comparison of sensitive methods. J Chromatogr B Biomed Sci Appl 2:277–283.

Fisher ND, Hughes M, Gerhard-Herman M, Hollenberg NK. 2003. Flavanol-rich cocoa induces nitric-oxide-dependent vasodilation in healthy humans. J Hypertens 21:2281–2286.

Heiss C, Dejam A, Kleinbongard P, Schewe T, Sies H, Kelm M. 2003. Vascular effects of cocoa rich in flavan-3-ols. JAMA 290:1030–1031.

Hensley K, Robinson KA, Gabbita SP, Salsman S, Floyd RA. 2000. Reactive oxygen species, cell signalling, and cell injury. Free Radic Biol Med 28:1456–1462.

Holt RR, Lazarus SA, Sullards MC, et al. 2002. Procyanidin Dimer B2 (epicatechin-(4β-8)-epicatechin) in human plasma after the consumption of a flavanol-rich cocoa. Am J Clin Nutr 76:798–804.

Knekt P, Kumpulainen J, Jarvinen R, et al. 2002. Flavonoid intake and risk of chronic diseases. Am J Clin Nutr 76:560–568.

Kris-Etherton PM, Lefevre M, Beecher GR, Gross MD, Keen CL, Etherton TD. 2004. Bioactive compounds in nutrition and health-research methodologies for establishing biological function: the antioxidant and anti-inflammatory effects of flavonoids on atherosclerosis. Annu Rev Nutr 24:511–538.

Kuhnle G, Spencer JP, Schroeter H, et al. 2000. Epicatechin and catechin are O-methylated and glucuronidated in the small intestine. Biochem Biophys Res Commun 277:507–512.

Lecomte MC, Boivin P. 1981. Filterability and pharmacology. Effects of methylxanthine derivatives on red cell phosphorylation. Scand J Clin Lab Invest Suppl 156:291–295.

Liu Z-Q, Luo X-Y, Sun Y-X, Chen Y-P, Wang Z-C. 2002. Can ginsenosides protect erythrocytes against free-radical-induced hemolysis? Biochim Biophys Acta 1572:58–66.

Lotito SB, Actis-Goretta L, Renart ML, et al. 2000. Influence of oligomer chain length on the antioxidant activity of procyanidins. Biochem Biophys Res Commun 276:945–951.

Mabile L, Piolot A, Boulet L, et al. 2001. Moderate intake of n-3 fatty acids is associated with stable erythrocyte resistance to oxidative stress in hypertriglyceridemic subjects. Am J Clin Nutr 74:449–456.

Mackenzie GG, Carrasquedo FA, Delfino JM, Keen CL, Fraga CG, Oteiza PI. 2004. Catechin, epicatechin and dimeric procyanidins inhibit PMA-induced NF-κB activation at multiple steps in Jurkat cells. FASEB J 18:167–169.

Maron DJ. 2004. Flavonoids for reduction of atheroscelerotic risk. Curr Atheroscler Rep 6:73–78.

Mennen LI, Sapinho D, de Bree A, et al. 2004. Consumption of foods rich in flavonoids is related to a decreased cardiovascular risk in apparently healthy French women. J Nutr 134:923–926.

Miki M, Tamai H, Mino M, Yamamoto Y, Niki E. 1987. Free-radical chain oxidation of rat red blood cells by molecular oxygen and its inhibition by α-tocopherol. Arch Biochem Biophys 258:373–380.

Morena M, Cristol J, Canaud B. 2000. Why hemodialysis patients are in a prooxidant state? What could be done to correct the pro/antioxidant imbalance. Blood Purif 18:191–199.

Moriguchi T, Takasigi N, Itakura Y. 2001. The effects of aged garlic extract on lipid peroxidation and the deformability of erythrocytes. J Nutr 131:1016S–1019S.

Morton LW, Caccetta RA, Puddey IB, Croft KD. 2000. Chemistry and biological effects of dietary phenolic compounds: Relevance to cardiovascular disease. Clin Exp Pharmacol Physiol 27:152–159.

Natsume M, Osakabe N, Oyama M, et al. 2003. Structures of (-)-epicatechin glucuronide identified from plasma and urine after oral ingestion of (-)-epicatechin: Differences between human and rat. Free Radic Biol Med 34:840–849.

Niki E, Komuro E, Takahashi M, Urano S, Ito E, Terao K. 1988. Oxidative hemolysis of erythrocytes and its inhibition by free radical scavengers. J Biol Chem 263:19809–19814.

Orozco TJ, Wang JF, Keen CL. 2003. Chronic consumption of a flavanol- and procyanidin-rich diet is associated with reduced levels of 8-hydroxy-2′-deoxyguanosine in rat testes. J Nutr Biochem 14:104–110.

Pearson DA, Paglieroni TG, Rein D, et al. 2002. The effects of flavanol-rich cocoa and aspirin on *ex vivo* platelet function. Thromb Res 106:191–197.

Sadik CD, Sies H, Schewe T. 2003. Inhibition of 15-lipoxygenases by flavonoids: Structure-activity relations and mode of action. Biochem Pharmacol 65:773–781.

Sagara M, Kanda T, NJelekera M, et al. 2004. Effects of dietary intake of soy protein and isoflavones on cardiovascular disease risk factors in high risk, middle-aged men in Scotland. J Am Coll Nutr 23:85–91.

Sanchez-Rabaneda F, Jauregui O, Casals I, Andres-Lacueva C, Izquierdo-Pulido M, Lamuela-Raventos RM. 2003. Liquid chromatographic/electrospray ionization tandem mass spectrometric study of the phenolic composition of cocoa (Theobroma cacao). J Mass Spectrom 38:35–42.

Sesso HD, Gaziano JM, Liu S, Buring JE. 2003. Flavonoid intake and the risk of cardiovascular disease in women. Am J Clin Nutr 77:1400–1408.

Sharma SC, Sharma S, Gulati OP. 2003. Pycnogenol prevents haemolytic injury in G6PD deficient human erythrocytes. Phytother Res 17:671–674.

Steinberg FM, Bearden MM, Keen CL. 2003. Cocoa and chocolate flavonoids: Implications for cardiovascular health. JADA 103:215–223.

Sugiyama M, Fung KP, Wu TW. 1993. Purpurogallin as an antioxidant protector of human erythrocytes against lysis by peroxyl radicals. Life Sci 53:39–43.

Takahashi T, Kamiya T, Hasegawa A, Yokoo Y. 1999. Procyanidin oligomers selectively and intensively promote proliferation of mouse hair epithelial cells *in vitro* and activate hair follicle growth *in vivo*. J Invest Dermatol 112:310–316.

Taubert D, Berkels R, Roesen R, Klaus W. 2003. Chocolate and blood pressure in elderly individuals with isolated systolic hypertension. JAMA 290:1029–1030.

Verstraeten SV, Keen CL, Schmitz HH, Fraga CG, Oteiza PI. 2003. Flavan-3-ols and procyanidins protect liposomes against lipid oxidation and disruption of the bilayer structure. Free Radic Biol Med 34:84–92.

Wan Y, Vinson JA, Etherton TD, Proch J, Lazarus SA, Kris-Etherton PM. 2001. Effects of cocoa powder and dark chocolate on LDL oxidative susceptibility and prostaglandin concentrations in human. Am J Clin Nutr 74:596–602.

Zhang A, Zhu QY, Luk YS, Ho KY, Fung KP, Chen ZY. 1997. Inhibitory effects of jasmine green tea epicatechin isomers on free radical-induced lysis of red blood cells. Life Sci 61:383–394.

Zhu QY, Huang Y, Tsang DSC, Chen ZY. 1999. Regeneration of alpha-tocopherol in human LDL by green tea catechins. J Agric Food Chem 47:2020–2025.

Zhu QY, Holt RR, Lazarus SA, Orozco TJ, Keen CL. 2002. Inhibitory effects of cocoa flavanols and procyanidin oligomers on free radical-induced erythrocyte hemolysis. Exp Biol Med 227:321–329.

Zou CG, Agar NS, Jones GL. 2001. Oxidative insult to human red blood cells induced by free radical initiator AAPH and its inhibition by a commercial antioxidant mixture. Life Sci 69:75–86.

